# Expanded transition spaces: the case of Garrwa

**DOI:** 10.3389/fpsyg.2015.00251

**Published:** 2015-03-10

**Authors:** Rod Gardner, Ilana Mushin

**Affiliations:** ^1^School of Education and Professional Studies, Griffith Institute for Educational Research, Griffith UniversityMount Gravatt, QLD, Australia; ^2^School of Languages and Cultures, University of QueenslandQLD, Australia

**Keywords:** conversation analysis, transition spaces, turn-taking, Aboriginal conversation, conversation and culture

## Abstract

Accounts of turn-taking in much of the CA literature have largely focused on talk which progresses with minimal gaps between turns at talk, longer gaps being found to be symptomatic of, for example, engagement in non-talk activities, or as indicators of some kind of trouble in the interaction. In this paper we present an account of turn-taking in conversations between Indigenous Australians where longer gaps are frequent and regular. We show that in sequences of such slow-paced conversation, gaps are not always treated as problematic, nor are they associated with non-talk activities that might inhibit talk. In such contexts we argue that there is less orientation to gap minimization, reflecting a lack of pressure for continuous talk. We also discuss qualitative differences in the nature of the gaps between turns in which there is a selection of next speaker, and those where no next speaker has been selected. Finally we consider whether such talk is a feature of Indigenous Australian conversation, or a more widespread practice.

## Introduction

This report had its genesis in a project investigating Aboriginal Australian conversation in the Garrwa language, with a focus on turn-taking practices (Gardner and Mushin, 2007; Mushin and Gardner, 2009, 2011; Gardner, 2010). One observation that emerged from this study was that the pace of these conversations appeared overall to be slower than has been reported for conversation in most of the literature on turn-taking. A specific feature of this slowness was seen in the distribution of inter-turn gaps of silence—the time it took for a next speaker to begin talking when the conversational floor was free. Ethnographic reports of Australian Aboriginal conversation have suggested a tolerance for greater gaps between turns than for Anglo Australian conversation (Walsh, 1991; Eades, 2000, 2007). This prompted the question whether this perceived slower pace indicated something about the fundamental ways in which Aboriginal Australians conduct their conversations, particularly in the overall pace of the talk and timing of speaker change. One hypothesis for these extended gaps between turns is, as Walsh (1991) implied, that Aboriginal Australians are orienting to a different set of rules for conversational turn-taking than are found in those societies that have been the basis of most investigations of turn-taking phenomena, starting with Sacks et al.'s (1974) seminal paper on the systematics of turn-taking using English language data. Walsh suggested that Aboriginal talk is “broadcast” to all present rather than to specific recipients, and further, that in a “non-dyadic” mode of speaking. He also claimed that Aboriginal speakers have a much greater tolerance for silence in conversation.

An alternative hypothesis is based on what Schegloff (2007) has posited as possible departures from interactional formats familiar to Western industrialized nations (which) involve what might be called “differences in the value of variables.” Under this hypothesis, the basic rules for turn-taking are the same, but the lengths of time that count as silence may be calibrated differently across cultures. This suggestion has received support from a study by Stivers et al. (2009), which examined the delays (or “response offsets”) in responding to polar questions across 10 languages from five continents that were different in terms of language family and culture. They established that longer delays in response were associated with four parameters: if a question was not answered rather than answered; if a question was disconfirmed rather than confirmed; if a response was verbal rather than visible (such as a head nod); and if the speaker had no gaze contact with the recipient rather than gaze contact. They found a mean response time of just over 0.2 s, but variation across languages, with Japanese the shortest at less than 0.1 s, and Danish the longest at a little under 0.5 s. The overall mode was 0 s (supporting the Sacks, Schegloff, and Jefferson claim reported below that turn transitions with no gap and no overlap are common), with a mode variation between 0.0 and 0.2 s across the 10 languages. They found that in all languages the response time was between zero and half a second, which they argue suggests a universal across all languages for gap minimization. They found no correlations of length of delays between closely related languages nor between similar cultures. The variations that they found in response offset times arose from what they termed “a different cultural “calibration” of delay” (p. 10590), which, they claim, has to do with the general pace of conversations and the general tempo of life in the communities in which these languages are spoken.

What we report in this paper is that that these Garrwa speakers have tolerance for silences between turns that appears to be greater than it is for English conversation, including Anglo-Australian conversation, and indeed there is some evidence (cf. Gardner, 2010) that this appears to be stronger than for any of the 10 languages reported in Stivers et al. (2009). However, we provide evidence that in some Anglo-Australian conversations, under certain situational conditions, there also appears to be a greater tolerance for lengthy interturn silences. In these conversations, couples were at home alone in the evening, engaging in “non-focused” talk (cf. Couper-Kuhlen, 2010). This leads us to the hypothesis that what we are finding is not any difference in the ways in which speakers allocate turns of talk, but rather that the circumstances in which the talk is occurring may be what is leading to “differences in the values of variables,” in this case the length of interturn silences. We also find strong evidence that Aboriginal speakers of Garrwa generally adhere to the rules of turn-taking as proposed by Sacks et al. (1974).

Based on these observations, the questions we address in this paper are whether delayed onset of talk by a next speaker:

- constitutes a different system of turn-taking among Garrwa from that outlined in Sacks, Schegloff, and Jefferson;
- can be accounted for within the existing parameters for turn-taking without resorting to cross-cultural explanations.

Sacks et al. (1974) proposed a set of rules that derive from two proposals: first, that a turn at talk is made up of a word, phrase or sentence that can stand alone and make full sense in the context of the conversation (a “turn constructional unit” or TCU)^1^, and second, that there is a short period of time at the end of a TCU within which change of speaker is warranted by the rules (the “transition relevance place” or TRP). Where a “normal” transition-relevance place begins and ends is not often discussed explicitly in the literature, but the normal, or default space has been declared to be one “beat of silence,” i.e., the time it takes to say a single syllable at normal rate (Jefferson, 1984; Schegloff, 2000). Wells and Macfarlane (1998) suggest that it extends from a final, turn-ending projecting accent in a TCU to the onset of a next speaker's talk, which may typically be about two beats, or 0.2 s^2^. If someone starts speaking outside the TRP, this can be treated by participants as problematic.

There are two rules, which specify how change of speaker occurs. The first of these is divided into three parts. The first of these, rule 1a, states that if in the course of a turn the speaker of that turn selects someone to speak next, for example by naming them, by gaze, by touch or by asking a question that only one other participant has the knowledge to answer. Under such circumstances, the speaker who has been selected is obliged to begin speaking at the TRP that occurs at the end of the TCU that is in progress. The second part, rule 1b, comes into play if the current speaker does not select a next speaker, and states that at the next TRP, any speaker other than the current speaker may start speaking (or may “self-select,”) and if there is more than one other participant, the first to start has rights to the floor. The third part, rule 1c, states that if no other speaker self-selects under rule 1b, then the current speaker may (but need not) continue speaking, thereby producing a second TCU in their turn. A second rule is necessary because under rule 1c, there has been no change of speaker, so in order to state how such change occurs, Sacks, Schegloff, and Jefferson made explicit that if current speaker does continue under rule 1c, then the three parts of the first rule are recycled until change of speaker does occur.

This simple set of rules has a power that may not be immediately obvious, namely that, first, the system is built to ensure that speaker change occurs frequently, but also, second, bids for speakership need to be made with precision timing that requires preparation for the bid while the current TCU is still underway.

First we demonstrate that the participants in these Garrwa conversations on many occasions allocate turns very much in the way predicted by Sacks et al. (1974). Then we examine some examples of talk with expanded transition spaces, in which gaps between turns appear to be longer that Sacks, Schegloff, and Jefferson talk of as a normal TRP. They claim as one of their “gross observations” that speaker transitions with no gap or overlap are common, and together with transitions with slight gaps or overlaps, they make up the “vast majority of transitions” (p. 701). In the Garrwa conversations, we commonly find a tolerance for silence between turns of up to several seconds. Sometimes these silences can be accounted for by non-talk activities, such as drinking or grooming that may be distracting the speaker from the ongoing talk, but some activities, such as grooming, do not in and of themselves disable the ability to talk. We then examine whether there are differences in the length of long silences following current speaker selection of the next speaker (rule 1a) or following self-selection of next speaker (rule 1b), and we report that we do find regular differences in the length of the long silences after application of rule 1a, compared with turns after application of rule 1b. Finally, we pose the question of the degree to which these unusually long silences are a phenomenon of Aboriginal Australian interactions—and perhaps the interactions of some other indigenous peoples (cf. Scollon and Scollon, 1981; Hoymann, 2010)—or a more general practice of talk-in-interaction that derives from a lack of pressure for continuous talk, associated with situational factors such as intimates just “hanging out” with nothing particular to talk about, in familiar surroundings with no pressure to “get things done.” We then begin to examine the extent to which long gaps between turns are a cultural phenomenon by examining some examples of long gaps between turns in two Anglo-Australian English language conversations.

A number of Conversation Analysts have suggested that delayed responses are regularly associated with problems in the talk. There may be talk-internal reasons for the silences, such as word searches (Goodwin and Goodwin, 1987; Hayashi, 2003), attempting to gain the attention of another speaker (Goodwin, 1981), speech impairment of one or more of the participants (Goodwin, 1995b). Other delays may occur prior to dispreferred, or non-agreeing responses (Pomerantz, 1984a), or during resistance to requests (Davidson, 1984)^3^. Pomerantz (1984b) points to recipient problems due to unclear references in the prior speaker's turn, a lack of recipient knowledge that the prior speaker had assumed, and recipient disagreement to account for silences of up to about a second between a first pair part (such as a question) and a second pair part (such as an answer). Davidson (1984) notes that silence after a first pair part can be a result of “puzzlement, or lack of clarity about exactly what's being offered” (p. 127), as well as difficulty in hearing and doubt about the acceptability of the proposal (p. 103). In some forms of institutional talk, long silences may be tolerated, for example in language (and perhaps other) classrooms where a teacher may wait a long time for a student answer to her question (Gardner, 2007). Silences after first pair parts can be precursors to a “potential rejection” (p. 103). As Jefferson (1986) puts it, ‘for the vast majority of cases “utterance + pause” does not capture the routine ways that recipients monitor talk in progress. What it does recurrently catch is a particular sort of problem posed for speakers’ (p. 179). When these problematic silences occur, she also notes that they tend not to exceed about 1 s in length (Jefferson, 1989). Longer silences are avoided: “Whatever one might mean by “waiting long enough,” waiting beyond 1 s is waiting too long” (Jefferson, 1986, p. 179).

There was, however, no indication that most of the silences in the Garrwa conversations were “problematic for the participants” (Jefferson, 1989, p. 170), which is in contrast to most of the longer silences reported in Jefferson's “standard maximum silence” paper. There was little evidence that these kinds of silences were particularly “meaningful.” They appeared to be indicators of normal conversation.

Thus, in order to determine whether the talk is truly slow-paced, and unusually long gaps are regularly occurring, possibilities such as problems of various kinds, or orientation to another activity, need to be ruled out. In fact, some of the silences in the conversations studied for this project could be explained by interactional features of these kinds. Many others, however, could not. In the sequences of slow-paced talk we are describing, gaps are the norm, and there is little evidence of anything problematic or unusual in the talk. In fact, even where the slow-paced talk occurs around “problematic” activities, the talk is conducted even more lethargically than similar situations reported in the literature, with regular gaps far exceeding Jefferson's “one second” metric.

### Rules, gaps, and lapses

The focal point for this paper is the nature of gaps of silence between turns, and the interesting case is what happens if current speaker does not continue, as this is the point at which gaps between turns emerge (cf. Wilson and Zimmerman, 1986). As Sacks et al. (1974) state, these turn-taking rules have a number of consequences for the conduct of ordinary conversation, including an orientation to the minimization of gaps between turns at talk. For example, if self-selection is used, then the incoming speaker is constrained by the possibility of current speaker continuing under 1c, as well as possible competition from other self-selectors, so an early start—as early as possible in the TRP—is necessary to assure speakership. As Moerman (1988) puts it, ‘there is some pressure upon a person who wants to speak next to come in a little before … a possible end. Moreover, if he doesn't come in *now*, he may not get to come in *next* (and) an aspiring speaker who doesn't get to have his say *next*, might *never* get to have it’ (p. 20). In a similar vein, Fox (2007) notes that ‘speakers and recipients in real-time conversation have immense time pressures on them … recipients must be ready to start up a turn which is in some way responsive to the current turn, without delay, as soon as the speaker has come to possible completion of current turn’ (p. 314).

As Sacks et al. (1974) say, ‘The components and the rule set, in organizing transfer exclusively around transition-relevance places, provide for the possibility of transitions with no gap and no overlap’ (p. 708). Notwithstanding this provision, and Moerman's and Fox's observations above, silences can occur, and it is the very optionality of rules 1b and 1c—no speaker is obliged to self-select, nor is a current speaker obliged to continue if no other self-selects—that allows for the possibility of silences that can grow into extended gaps, and ultimately into lapses in the conversation, which is when participants disengage from each other. As Sacks, Schegloff and Jefferson put it, ‘discontinuities occur when, at some transition-relevance place, a current speaker has stopped, no speaker starts (or continues), and the ensuing space of non-talk constitutes itself as more than a gap—not a gap, but a lapse’ (p. 714). Note also that lapses can only occur after a current speaker has chosen not to continue when no other speaker has self-selected. No lapse can properly occur under the “current speaker selects next” provision^4^. As the authors point out, in such an event, ‘a silence after a turn in which a next has been selected will be heard not as a lapse's possible beginning, nor as a gap, but as a pause before the selected next speaker's turn-beginning’ (p. 715)^5^. In contrast, if no next speaker is selected, and no other speaker self-selects, and further, current speaker elects not to continue under 1c, then “a series of rounds of possible self-selection by others and self-selection by current to continue—rules 1b and 1c—may develop, in none of which are options to talk exercised, with the thereby constituted development of a lapse in the conversation” (p. 715) The implication of this is that in turn-by-turn talk participants are under considerable pressure to produce their turns early, within the transition space, but there are also provisions in the rules for turns to be delayed, which ultimately can account for lapses in a conversation.

## Data and methods

Our corpus consists of five conversations recorded in two remote Aboriginal communities in Australia's Northern Territory, near the Gulf of Carpentaria. Four conversations were recorded in the small town of Borroloola, which has a population of about 1000, the vast majority of whom are Indigenous Australians belonging to four different language groups. These conversations were audio recorded only^6^. The fifth conversation was recorded at Robinson River, a Garrwa (Aboriginal) community with a population of about 250 about 2 h drive south of Borroloola. There were five principal participants in these conversations: two elderly Garrwa women in Borroloola (Tina and Ellen^7^), and three elderly Garrwa women in Robinson River (Daphne, Hilda, and Katelin). The Borroloola data were mostly recorded on the veranda of a cabin, the second author and occasional passers-by entered the conversations on a few occasions. The Robinson River data features three elderly Garrwa women who were sitting on the ground on the front porch of the house of one of the women. We call this the “Porch” data. The recording, which lasts for over 2 h in total, was initially set up by the second author to record interactions between fluent Garrwa speakers and children who are not fluent in Garrwa. After about 20 min, the children leave (or are told to leave) by the elderly women, leaving the three of them alone on the porch. It is at this point that the task of recording Garrwa language ceases to be the focus of the talk and the topics turn to matters such as planning future hunting expeditions, complaining, reminiscing, and interacting with other residents as they pass by.

In all of the conversations much of the talk a mix of Garrwa, a local variety of Kriol (a local creole language) and Aboriginal English, reflecting normal patterns of community multilingualism. A total of about 35 min of the Borroloola conversation, and about 26 min of the Porch conversation have been closely transcribed. In the collection of extracts from these conversations we had over 400 speaker transitions, and within this collection we analyzed more closely about 160 of the longer gaps. Four of the five women acted as informants and teachers of the language as part of the transcription process.

Where we have video evidence, most (but not all) of the long silences do not occur when participants are engaged in non-talk activities, such as those Jefferson (1989) points to explain the extended silences in her data (including examining a train timetable, scanning the surroundings in a neighborhood block party, or writing down an address). On some occasions in our data, something is going on in the environment that might hold the participants' attention, but on others the speakers are sitting around doing nothing apart from talking.

We use Conversation Analysis to analyse the silences within sequences of talk (Sidnell, 2011; Sidnell and Stivers, 2012). These elderly women were among those who taught the second author the grammar of Garrwa, and features of morphology are included in the transcriptions to show turn construction features. The women also provided us with some ethnographic and local contextual information where we needed it to understand what was happening in the interactions, for example about the collection of wild honey (“sugarbag”).

We measured the pauses by manually locating them through the inspection of waveforms in Audacity (Audacity Team, 2013), and these figures were then rounded up or down to the nearest tenth of a second. The response offset is measured between the last element of the first turn, and the first element of the responding turn, whether it be a particle such as *well* or *uhm*, or a lexical item. In breaths or clicks were also included as part of the second turn.

## Results

In the data we have examined for this paper, we commonly found stretches of talk such as extract 1.

(1) Porch:2.1:866:PD2.

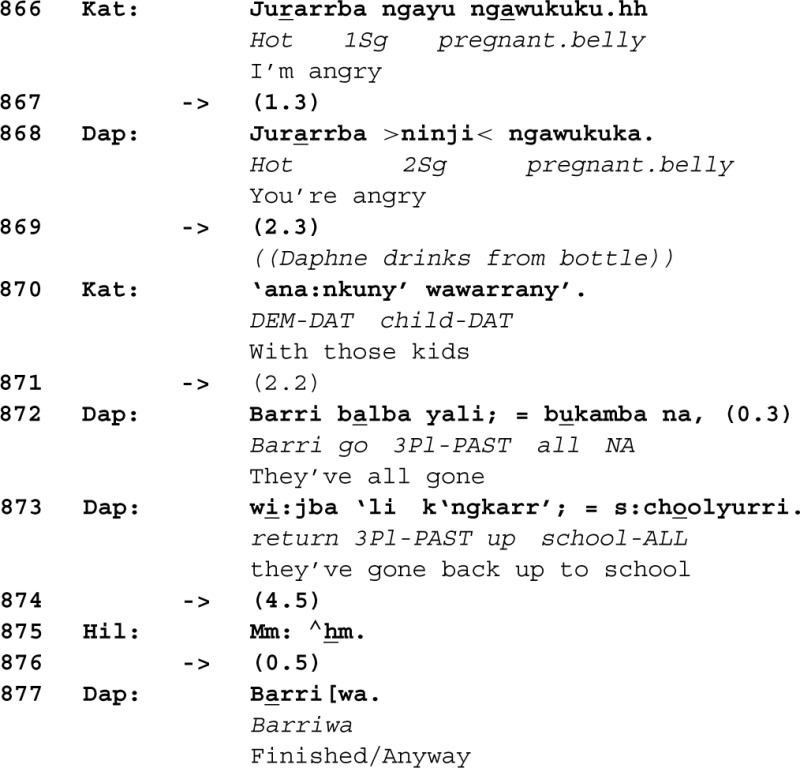



Extract 1 occurred at Robinson River while the three women were sitting quietly alone, mostly without eye contact, though occasionally turning to each other. Two of the women are sitting next to each other, facing the house at an angle. The third is sitting at an angle of about 90° to the other two, behind them and facing away from the building, and two (Katelin and Daphne) remained in these positions for the whole of the 2 h of the recording, with Hilda arriving after about 40 min. To obtain eye contact with each other, they needed to turn their heads 30° or more. The video for extract 1 shows Katelin fiddling with small unidentified objects in her lap, Daphne with a bottle of drink, and Hilda stroking a coolamon^8^. At times such activities lead to what Goodwin (1981, p. 106) has called “activity-occupied withdrawal” from the talk, by which he meant activities such as writing, preparing food, grooming, attending to equipment or other artifacts (Goodwin, 1981, 1994, 1995a). In the Borroloola and Porch conversations such activities mostly did not appear to disrupt the talk, occurring during both talk and silences. Some other activities, however, namely drinking, smoking, coughing and nose blowing, were potential talk inhibitors^9^.

Between each turn in this extract (except the last two), there are gaps of silence of between 1.3 and 4.5 s, and there is no evidence, either auditory or visual, that the participants experience any problem with the talk, nor are they engaged in other activities, apart from fiddling with small objects in their hands and, in line 869, during the 2.3 s pause in line 869, Daphne drinking from a bottle.

### Orienting to the rules of turn-taking

So what is happening here to the notion of orientation to TRPs and gap minimization? An initial point is that this has nothing to do with a different set of turn-taking rules. As Sidnell (2001) found for Caribbean Creole conversations, the turn-taking system in the conversations of these Garrwa speakers has the same fundamental organization as that described for American and British conversation. These women routinely speak when selected. They self-select when they are not selected. They sometimes respond with precision timing at TRPs, orienting to possible completion points of TCUs. In extract 2, Tina selects Ellen as next speaker by asking her a question (rule 1a). Ellen responds in “unmarked next position” (i.e., with one beat of silence) when she is asked a question by Tina.

(2) Garrwa3-.20.8.03:V3:37:0′30″

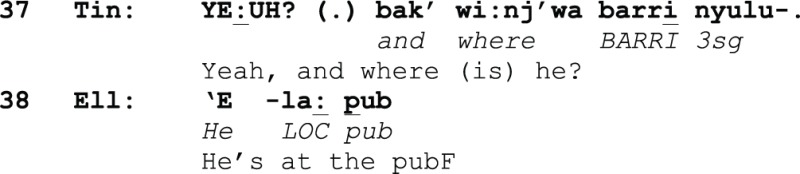



In extract 3, in line 60, Tina selects Ellen as next speaker under rule 1a, by initiating repair with “Who.” Ellen latches her response to this repair initiation. It can also be noted, however, that the gap between Ellen's question and Tina's repair initiation is almost twice as long as the average for English conversations (Kendrick, 2015).

(3) Garrwa3-10.10.03:V3:58:1′00″

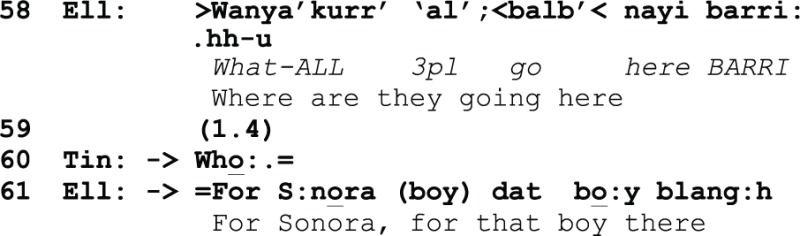



Self-selection under rule 1b can also occur with no gap. In extract 4, Tina announces that she had been dreaming, which does not select Ellen as a next speaker. Ellen then self-selects, on a non-related topic, latching her own remembering to Tina's turn.

(4) Garrwa:8.9.03-V3:18:0′25″

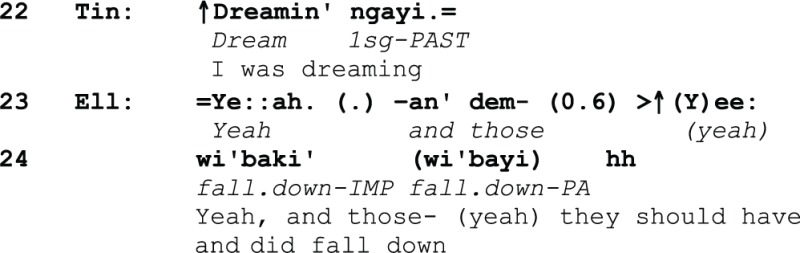



There are also cases of turn starts in terminal overlap, that is, early in the transition space. In extract 5, Daphne observes that two people are cooking a kangaroo, and Katelin notes how they are cooking it, overlapping with the last part of Daphne's turn.

(5) Porch2.8:614:0′30

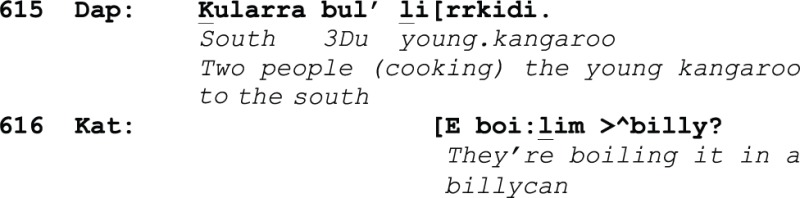



Another example of self-selection under rule 1b occurs in extract 6. In this example, in which they are talking to a man who is passing by, both Tina and Ellen self-select in 272 and 273, after 0.2 s, which is the later end of the transition space. Tina is first starter, which, under rule 1b, means she has rights to the floor. Ellen, the second starter, drops out, but then restarts (line 274) immediately Tina finishes her turn, latching her talk to the end of Tina's talk, just as predicted by the Sacks et al. (1974) turn-taking rules.

(6) Garrwa3:20.8.03:V3:268:5′05″

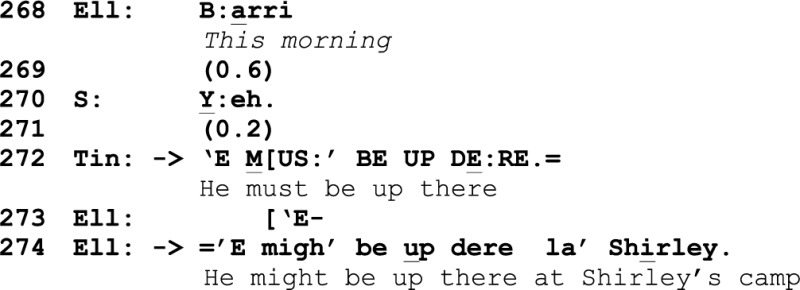



A further example of self-selection at a point of possible TCU completion occurs in extract 7, which is from the Porch data. Daphne is asking a passing girl to get her mother to bring some fish and chips. Daphne gets two responses to her request, one from the girl, and subsequently one from one of the other older women, Hilda.

(7) -Porch-2.10:1220:1′00″

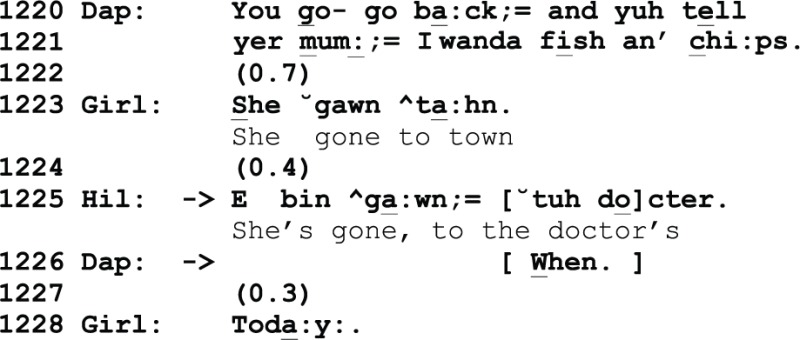



At a point when the second of these responses, by Hilda, is possibly complete, after “e bin gawn” (she's gone), Daphne asks a follow-up question, “when.”^10^ However, it turns out that there is more to come in Hilda's turn: “tuh docter” (to the doctor). This is an example of what Jefferson (1984) calls latched overlap, which occurs because Daphne has not predicted the extension of Hilda's turn.

In these conversations we also find examples of the occurrence rule 1c, in which a current speaker continues when no other speaker has self-selected under rule 1b. In extract 8, Katelin requests that the other two women start talking more as she has been doing most of the talking so far (this is for the benefit of the recording of the talk), as she is tired. There is no evidence in the video that she is directing her request at only one of the women through, for example, gaze selection. Katelin comes to a TRP at the end of line 846, with grammatical, intonational and pragmatic completion (cf. Ford and Thompson, 1996). There is no response within 0.4 s, at which point Katelin continues her turn with an account for her tiredness.

(8) Porch:2.8:845:3′50″

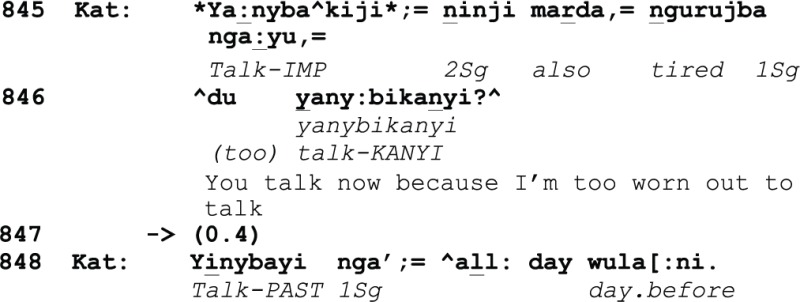



In extract 9, from one of the Borroloola conversations, we present two examples of rule 1c, in this case both clashing with 1b. This clash occurs because a self-selecting speaker comes in rather late—at the end or even beyond the “normal” transition space—at the same time as a current speaker elects to continue.

(9) Garrwa3:20.8.03:94:1′40″

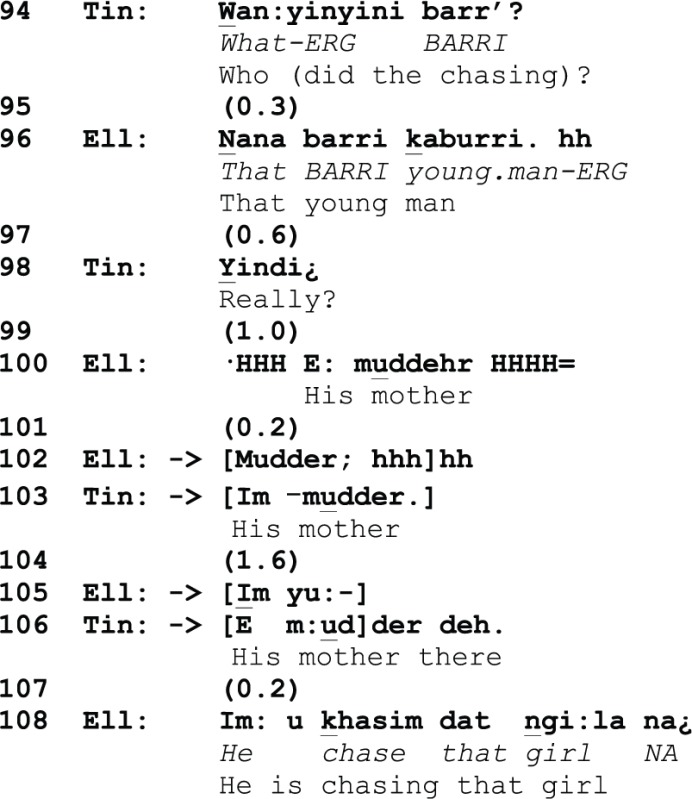



In line 98, Tina produces a newsmarker, which appears to be seeking confirmation that it was the young man who did the chasing. In response, Ellen makes reference to his mother^11^. After 0.2 s, which would be at the end of the normal transition space for British and American conversation (Wells and Macfarlane, 1998), there is a simultaneous start with Ellen repeating “mudder,” and Tina producing a confirming repetition, “im mudder.” As these two turns were produced simultaneously, it is now equivocal who, at the simultaneous start that follows in 102-3, is current speaker and who is next: in effect both are current speakers, so both, after a long transition space of 1.6 s, elect to continue, Tina continuing the same sequence with “E mudder deh,” and Ellen moving on to something new. However, Ellen drops out, Tina completes her turn, and 0.2 s after Tina finishes, Ellen restarts the turn she had abandoned.

In the final example in this section, extract 10 presents a sequence that can clearly be seen as potentially problematic, in the sense that a question is asked that never gets answered. However, there is no evidence from the talk or from the video that the participants orient to it as particularly problematic, not even Hilda, who asks the question.

(10) Porch2.7:545:4′30″

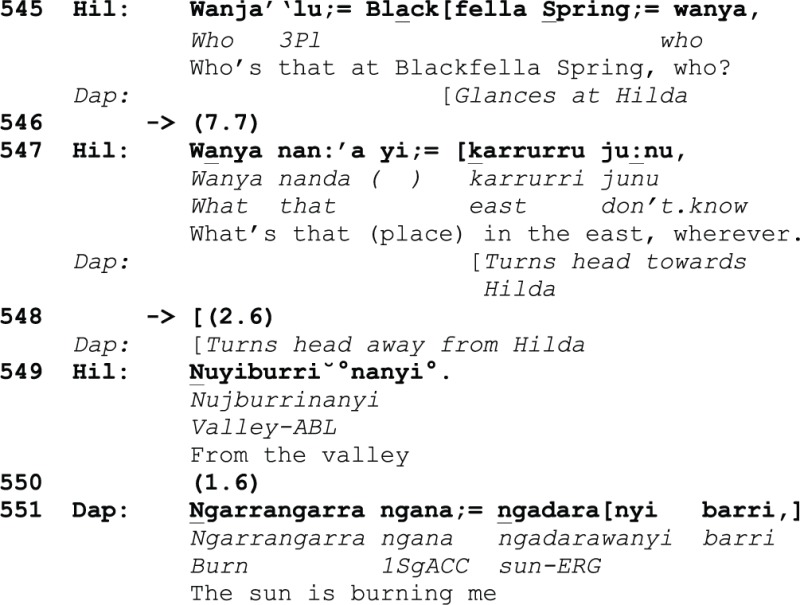



Hilda twice pursues an answer, following the rule 1c according to which if no other speaker self-selects then current speaker may continue. This happens in lines 547 and 549, but after these two attempts, she gives up. Throughout this extract, Hilda is gazing to her right at 40° toward Daphne, while the latter is fiddling with the cap on her bottle of soft drink, and the sound of gas escaping is audible. This suggests she may be preoccupied, (as is Katelin—not the addressee—who is brushing her hair). However, fiddling with a bottle cap is not an activity that would necessarily inhibit Daphne from answering Hilda's question. After the first question, Daphne glances at Hilda, but there is no response during a gap of 7 s. There is also evidence that Daphne has heard the second question, because toward the end of line 547, she turns her head toward Hilda, holds her gaze briefly, and then returns it to her drink bottle during the 2.6 s silence that follows. Then there is increment to this question in line 549, “Nuyiburri nanyi” (*from the valley*) and again nothing for 1.1 s, which is when Daphne does produce a turn, which, however, is not a response to the question, but a complaint about sitting in the sun. It is also notable that Hilda does nothing to show that she finds the lack of an answer to her question problematic, apart from twice pursuing the answer before dropping it.

These examples demonstrate that these Garrwa women can, and regularly do (though as extract 10 shows, not invariably), orient to projectably complete units of talk, the turn-taking rules, and transition places, as explicated in Sacks et al. (1974). What, then, is going on when there are regular long silences between turns in these conversations?

### Accounting for long silences and expanded transition spaces

We have reported so far that in our data longer gaps between turns are common. There are, however, striking differences in the length of silences between the “current-speaker-selects-next” (1a) and “next-speaker-self-selects” (1b) techniques. This may not be surprising, but this is another feature that shows that Garrwa speakers are conducting their conversation in a similar way to English conversationalists. When a current speaker selects a next, the response tends to come relatively quickly, though with a longer delay than has been observed in other languages. In all of the examples examined from our Garrwa corpus, silences after speaker selection occur regularly up to 1.5 s (Mushin and Gardner, 2009). In contrast, when no next speaker has been selected, the gaps can be much longer. In this section, we examine some extracts in which current speaker selects next, and following that, we consider some cases in which no next speaker has been selected.

#### Silences after selection of next speaker

As was noted above, silences occurring when a current speaker selects the next are of a very different order to silences when the next speaker self-selects. According to Sacks et al. (1974), if a current speaker has selected the next speaker, there is an obligation for the selected participant to speak as early as possible at the next transition space. If no next speaker has been selected, then no such obligation exists.

On some occasions the TRP may be “expanded,” for example because of activity-occupied withdrawal, or a dispreferred response. However, on many occasions in the Garrwa conversations, when a current speaker has selected a next under rule 1a, there is a gap preceding even a preferred response. Regularly, but not always, these silences are “filled” with relevant gestures, such as head nods which precede the talk, and there is thus no delay in the response in such cases. On others, there is no talk-supporting activity. There is a delay of 1.4 s in extract 11 between the question and answer.

(11) Porch:2.1:1002:IR-4:1′35″

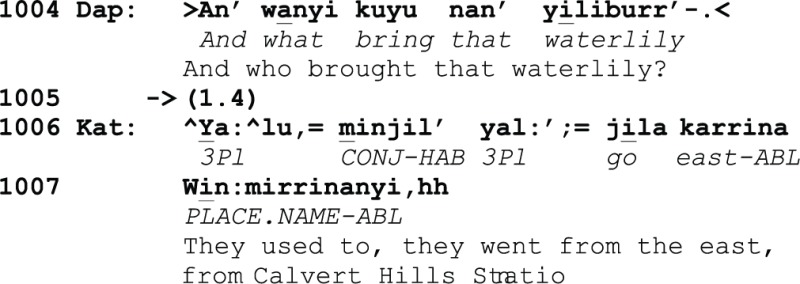



This is a case in which there is no gestural support of the talk. Katelin and Hilda are both looking at Daphne whilst she is asking the question. During the question and the 1.4 s silence, Katelin and Daphne have eye contact, though Katelin is fiddling with her collar, which is not an activity that necessarily precludes simultaneous talk. There is nothing in this sequence that indicates any trouble, nor does Katelin appear to have her attention on any other matter. The answer, when it comes, is preferred, though it is expanded (and it is expanded further beyond this short sequence)^12^. This contrasts with a typical preferred response in “Western” talk, where such a response tends to come quickly and briefly, without accounts or other expansions (Schegloff, 2007: 67ff).

The broader context of this adjacency pair is that it occurs during a reminiscing sequence about how in the old days they used to collect waterlilies to eat. This may help explain another feature of this sequence, namely that this is on the face of it an information-seeking question, which in English mostly attracts a brief, phrasal response (Fox and Thompson, 2010), but here we have an extended response—which is further extended beyond this adjacency pair (not shown), so this WH-question could be seen as a prompt for extending the reminiscing. Planning for an extended response may be a factor in the delay of 1.4 s.

Extract 12 is from the Borroloola corpus. There is no video to support the analysis, but this is included as an example of another delayed answer with a preferred response, with no perceptible trouble.

(12) Garrwa-9.10.03:V3:99:1′30″

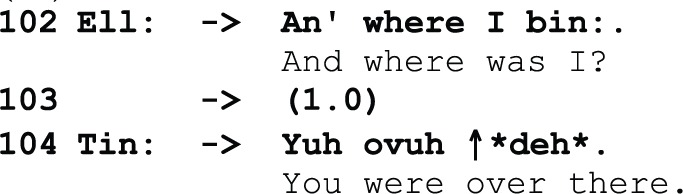



In extract 13, the silence cannot be accounted for even in part by non-talk activities or delays associated with a dispreferred response. Hilda repeats an answer that she had already provided once, namely that wild honey can be found at Hubblestrap. Daphne and Hilda have eye contact, with Daphne's neck “torqued” almost 90° toward Hilda (Schegloff, 1998). They are clearly focused on talking to each other.

(13) Porch2.10:1282:2′00″:IR-5:2″13″

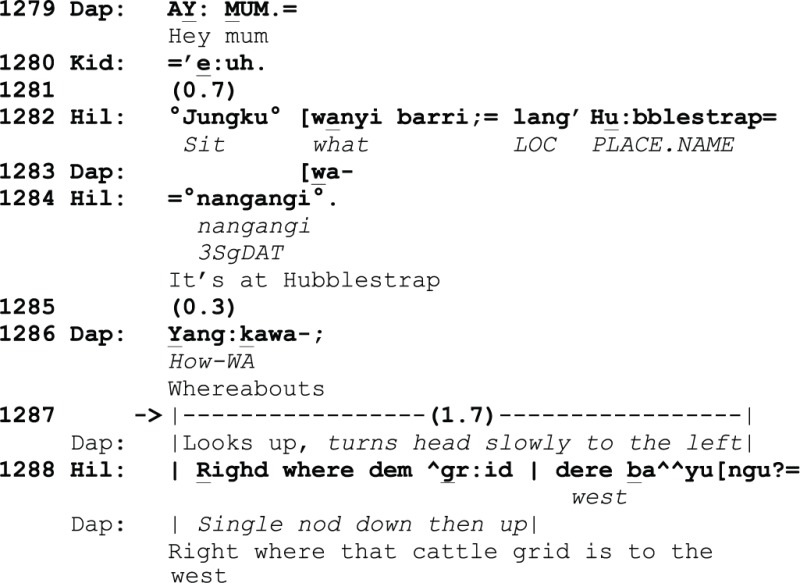



Between the answer that Hilda had given in 1225 in extract seven and this sequence, the three women had been talking to two boys who had arrived where they were sitting. Daphne then turns her head sharply toward Hilda in 1279 with a summons (the “Mum” in 1279 is a term of address directed at Hilda), which has the effect of Hilda repeating her answer, that the sugarbag is at Hubblestrap, and Daphne then asks for more specific information with “yangkawa” (whereabouts). There is a delay of 1.7 s between the repair initiation in 1286 and the response in 1288. What happens in this silence is fully oriented to the answer. Hilda turns her head slowly in a westerly direction (away from Daphne), and this takes up the whole 1.7 s, and then nods in that direction as she says “Righd where dem grid.” There is no hurry to start talking. The head turn prepares for the answer, and is accomplished prior to the verbal response. This languid response contrasts with the general practice in English (Goodwin and Goodwin, 1987) and Japanese (Kita and Ide, 2007) conversation, where it is most usual for the gesture and talk to occur very quickly after the prior turn, with the gesture slightly foreshadowing the words (Streek, 1993).

In the next extract Daphne urges the other two to hurry up so they can leave. Her directive in line 894 receives no immediate compliance from the other two. Indeed, first Hilda and then Katelin concur, but with substantial gaps of 1.2 and 2.4 s, respectively.

(14) Porch:2.8:894:4′40″:IR-3:4′12″

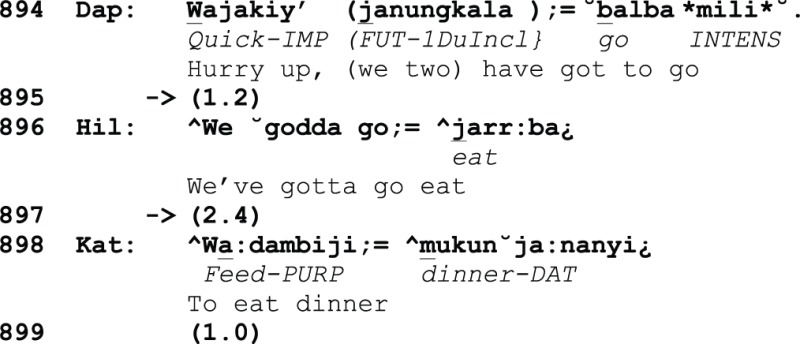



This is an agreeing sequence, that is, each turn is a preferred next: both recipients say they want to go to eat something, but neither shows any sign of complying with Daphne's directive by getting ready to leave. There is no observable ambient or interactional reason visible in the video for the delays between these turns. However, rather than the immediate or even early responses to preferreds that are reported in the literature (Levinson, 1983; Pomerantz, 1984a; Schegloff, 2007), there are substantial delays here. Daphne is waving away flies and then she picks up a small object and shakes it: but she does this *after* she has spoken. Hilda is stroking a coolamon throughout this sequence, but this is not an activity that interferes with the ability to talk. Daphne is urging them to quick action, so what better illustration of the slower pace of the talk could there be than this languorous hurrying up?

We can see from the examples presented in this section that sometimes when the current speaker selects next, the response occurs relatively promptly, mostly within about a second-and-a-half (see Mushin and Gardner, 2009 for a more detailed discussion), whilst others (not presented) have delays of less than a second. The metric with a maximum of about a second-and-a-half is similar to what Scollon and Scollon (1981, p. 25) claimed for Athabaskan. This metric is about half a second longer than Jefferson (1989) found for the American, British, and Dutch conversations she studied, where there was a “standard maximum silence” of about 1 s (0.9–1.2) for various kinds of silence. The longer silences she found could be accounted for by activities the participants were engaged in that interfered with the flow of the conversations. For these Garrwa conversations, there may be grounds for amending Jefferson's observation about waiting for 1 s to: “Whatever one might meant by “waiting long enough,” waiting beyond *one-and-a-half* seconds *after one has been selected* is waiting too long” [adapted from Jefferson (1986), p. 179]. In the next section, we shall discuss gaps of silence between turns where there is no selection of next speaker by the current speaker.

#### Silences before self-selection by next speaker

The metric of a maximum silence of one and a half seconds appears to apply only to turn transitions in which next speaker has been selected by current speaker, but not to self-selection in turn-by-turn talk. This can be partly explained by the fact that where there has been no selection of next speaker, there is no obligation for anyone to speak. A gap may ensue, and can extend until there is a lapse in the conversation and speakers disengage. In many cases in the Garrwa conversations, however, inter-turn silences of several seconds occur without any apparent orientation to a problem in the talk, nor any indication from changes in body posture that the conversation has lapsed. Some of these gaps can be explained in the same way as those discussed earlier: sometimes non-talk activities, or dispreferreds, or the ends of sequences and topic attrition occur in conjunction with longer silences. On other occasions, however, such factors do not appear to elucidate the silences.

In extract 15, which is from the beginning of the Porch conversation, there is very little non-talk activity from the three women: Katelin scratches her foot, and Daphne appears to wave away a fly, but otherwise they are sitting and looking mostly straight ahead, without eye contact. They do not appear to pay much attention to the barking dog, except for Hilda's question about it in line 8.

(15) Porch:2.6:001

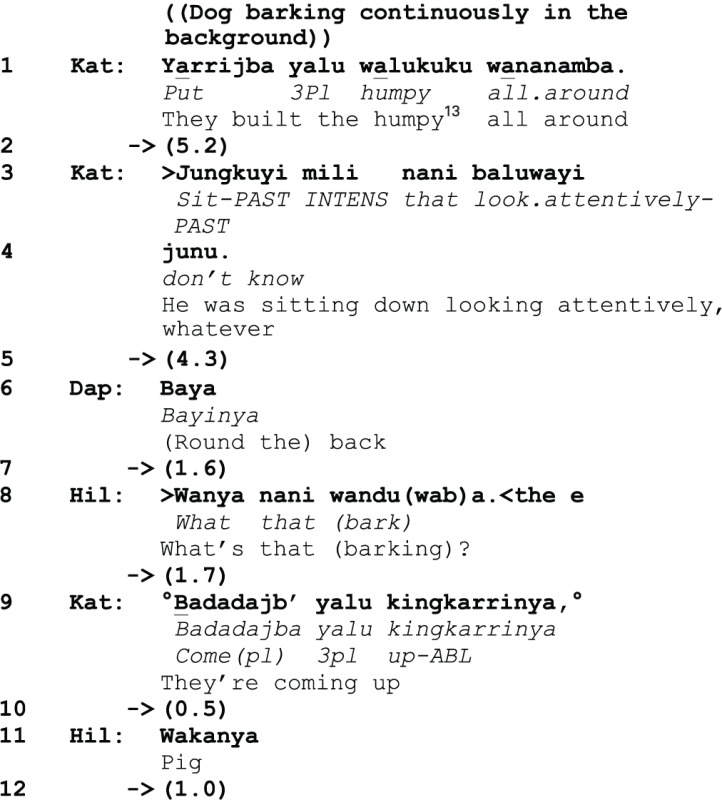



In the above extract, there is no topic that they are pursuing. They've been sitting around for a long time before this point in the conversation. There is no strong engagement. They move in desultory fashion from topic to topic, commenting on what is going on around them—a dog barking, a group of people approaching. Reference by Katelin to a “humpy” (temporary shelter) is followed by 5.2 s of silence. The talk in lines 3–4 is followed by a silence of 4.3 s. Daphne then self-selects with an observation that something is round the back. 1.6 s later, Hilda asks a question which does not get answered. 1.7 s after this, Katelin then observes and comments on some people approaching. Half a second later Hilda mentions the presence of a pig nearby.

In contrast to extract 15, where there is little topical continuity between the turns, in the next extract, the gaps—even the 7.6 s in line 115—are all between turns that are coherent self-selecting contributions in the flow of the talk.

(16) Garrwa2-9.10.03-V2:111: 1′45″

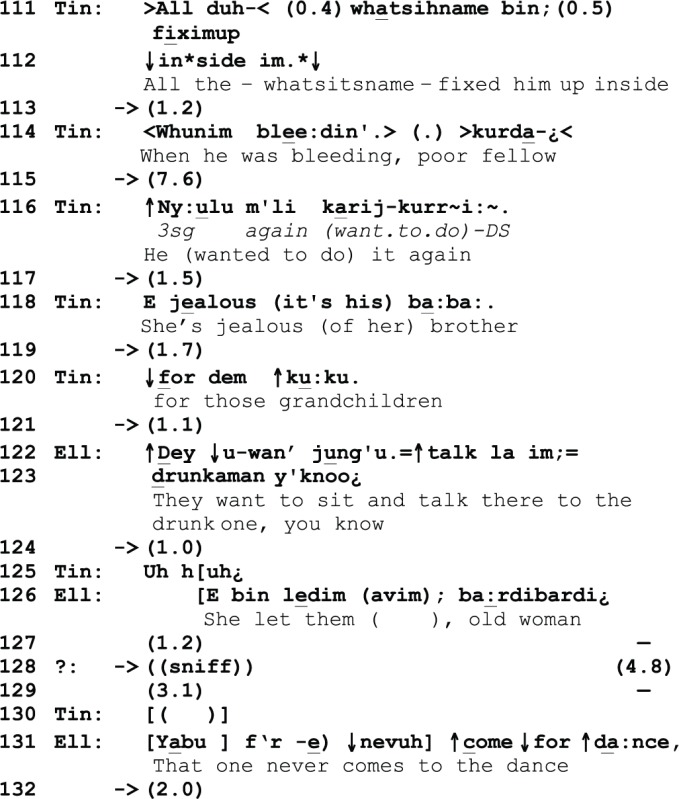



In the first part of this extract, all of the talk is by Tina, with no hearable responses from Ellen, although there appears to be no reason why Ellen could not have self-selected. This is not a storytelling, although Tina's first turns refer to a recent event in the town. They have been exchanging views about people. None of these turns selects a next speaker, there is no competition for the floor, no sense of having to get the next turn in “now and not later,” and neither participant shows any urgency in producing a next turn. Two of the silences could be analyzed as intraturn silences, namely the ones in line in line 113 and 119, each of which is an increment to Tina's prior talk. But even so, the prior talk in each case ends at a possible TRP, and thus speaker change is relevant. For the talk that comes after the 7.2 s silence in line 115, however, it is harder to analyse this as an intraturn silence, as what precedes it is potentially complete, and what follows is not an increment. The talk flows topically, and could have been produced as a coherent multi-unit turn without any silences. Furthermore, there is no aural evidence of any other activity during this silence.

In this section we have looked at inter-turn gaps that occur when no next speaker has been selected. We have found that there are numerous gaps in these positions, and some of them are very long. The extracts presented here are ordinary conversation, and some of the languor of the talk here can be explained by non-talk activity or dispreferred actions. However, most of these non-talk activities are grooming or fiddling with objects, activities that could accompany talk, in contrast for example, to reading or writing, which require more focused attention. In very many sequences in these conversations, the inescapable observation is that this talk is inherently languorous, and there is no attempt at minimization of gaps in the way that has been described for English conversation, for example in Sacks et al. (1974). Very long silences, or lapses, are not frequent, as we have only three silences of longer than 10 s in our corpus^14^. Furthermore, gaps of more than 2 or 3 s are not common. We do find overwhelming evidence of an orientation by these speakers to the rules of turn-taking as presented in Sacks, Schegloff, and Jefferson. The difference from most published literature on turn-taking is one of the “value of variables” (Schegloff, 2007, p. 74). It is a difference to one of Sacks, Schegloff, and Jefferson's gross observations: “transitions (from one turn to the next) with no gap and no overlap are common. Together with transitions characterized by slight gap or slight overlap, they make up the vast majority of transitions” (pp. 700–701). In many sections of these conversations, the majority of transitions are, in contrast, characterized by substantial gaps.

### Extended gaps in conversation in other contexts

An easy explanation of the phenomenon we are reporting in this paper would be one of cultural difference: that Aboriginal Australians have a different “conversational style.” Whilst we have found regular lack of gap minimization in these conversations, there is no evidence in the data of a different set of turn-taking rules for these speakers. As Sidnell (2001) noted in his study on Caribbean Creole conversations, “[t]here is, at time of writing, no empirical study which provides evidence that humans do conversation in a fundamentally different way” (1286). We provide no evidence for this either. Our study provides another example of a non-Western culture in which the fundamental organization of conversation appears to be “grounded in a species-specific adaptation to the contingencies of human social intercourse” (Sidnell, 2001, p. 1263). The difference is only that regularly there is a marked expansion of what counts, at least in Western conversation, as a normal transition place.

In fact, the slow pace of these conversations is not, on the evidence we have, culture–specific. We have some evidence that expanded transition spaces occur in “western” talk. In the data used by the first author for his work on response tokens (Gardner, 2001), there are examples of slow-paced conversations amongst Anglo-Australian couples who recorded themselves when they were at home alone. These are intimates, engaged in talk at times of day when the pressure is off, such as after the evening meal. There are times in these conversations that look and sound very similar to the Garrwa conversations. In extract 18, there are some very long gaps, including one of 4.3 s in line 163 following a question from Liz, that is, after she has selected Mel as next speaker.

(18) L&MC2ai-Languorous:144

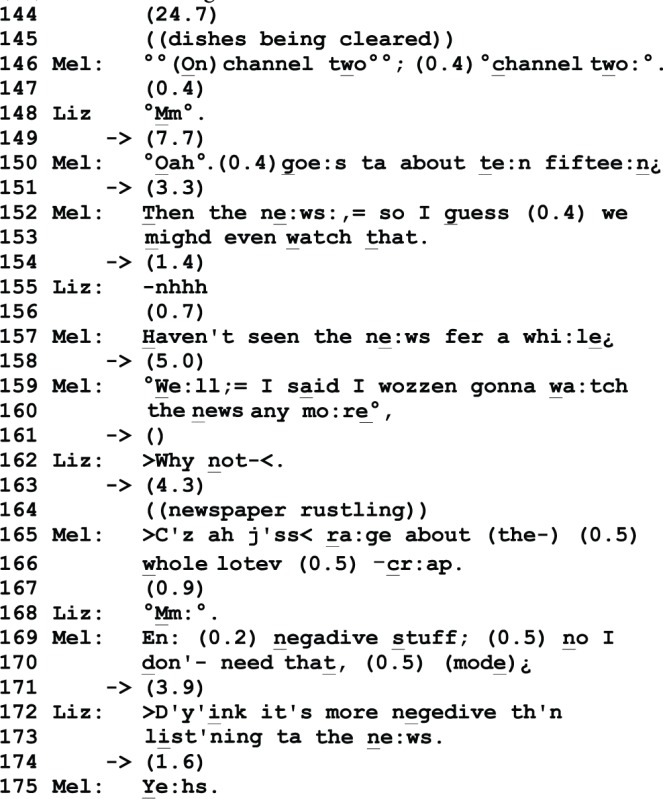



In the early part of this extract, Mel appears to be reading the television schedule for the evening in a newspaper, an example of “activity-occupied withdrawal,” with interspersed comments on programs. In the latter part, though, there are questions and answers, and the slow pace continues. The rustling newspaper in 164 might suggest that Mel is still engaged in reading—which would be an explanation for the long gap—but still, overall, the pace here is slow, and there is little attempt to minimize gaps.

In extract 19, Ike and Jan are driving, and are discussing who is going to have the car later.

(19) I&JW4a

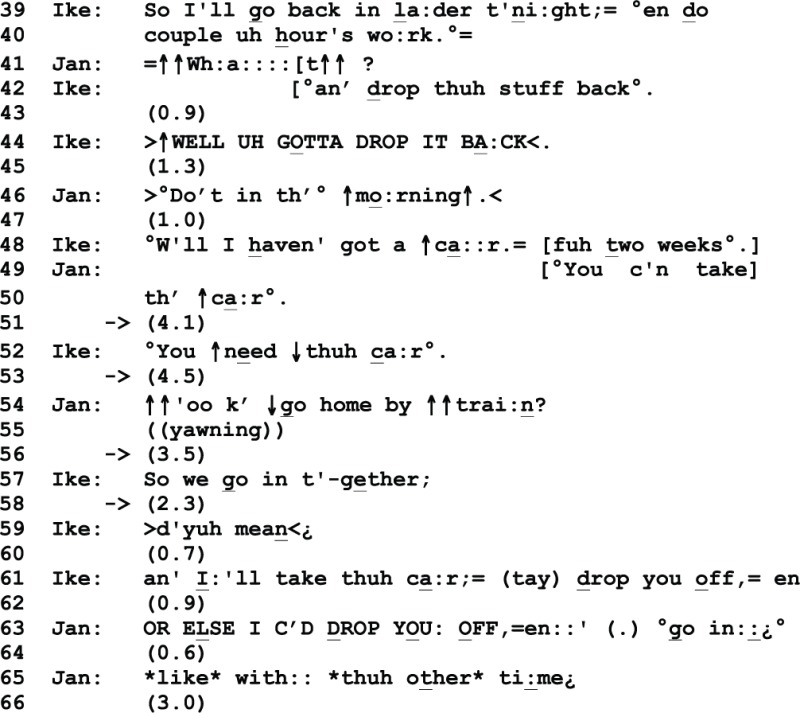



This conversation was recorded during a quite lengthy car journey. It begins with a proposal by Ike that he go into work in the evening, which gets a less than enthusiastic response from Jan. What follows is a series of proposals and counter-proposals and accompanying accounts. What is notable and relevant to this paper are the silences between turns in this sequence of up to 4 s.

The extended interturn silences of these Anglo-Australian conversations provide evidence that slow-paced talk is not restricted to cultures or societies such as the Garrwa people, or the Native American Warm Springs indigenous people that Philips (1983) reported on. We certainly have not found in these materials, as Philips (1976) claimed for the Warm Spring Indians, that turn-taking by their system was self-directed, or that anyone who wanted to speak did so and for as long as they wanted (Philips did not provide closely transcribed materials to back up her claim.). There are further situations in which talk characterized by expanded transition spaces is normal: second language classroom talk (from the first author's data), second language conversations (Wong, 2000), and perhaps most extremely in hypnosis sessions (Demosthenous, 2008). In the last of these, gaps of more than 10 s regularly occur between a hypnotist's question and a client's answer whilst in deep hypnosis.

What may be happening in these Garrwa conversations (and in the other interactions for which expanded transition spaces have been reported) is that a lack of gap minimization occurs with a greater frequency than has generally been reported in the Conversation Analysis literature. These interactions differ from those that have provided the data for many Conversation Analysis studies, in which the speakers are at dinner parties, are on the phone, are in animated groups engaged in lively discussion.

## Discussion

In the Stivers et al. (2009) study of turn-taking practices across 10 languages, key findings were that there is a general avoidance of simultaneous talk and a minimization of gaps between turns. However, they did find a variation in the length of average gaps of 0.25 s across these languages, leading them to conclude that the fundamental turn taking mechanisms are universal, with differences between languages being only quantitative. While the methods used for our study and its primary focus are different from theirs, and thus preclude any direct comparison, our findings do broadly support those of Stivers et al. Using the same corpus as for the current study, Mushin and Gardner (2009) noted that approximately 50% of silences in the Garrwa conversations were over 0.9 s, and Gardner (2010), also working with the same corpus, but focusing only on question-answer turns (*N* = 62), found the average gap between question and answer to be 0.75 s, about half as long again as the longest average silences found in a similar environment for any language in the Stivers et al. study^15^. These findings provide further support for the claim that there is some cultural variation in the timing of responses and of next turns generally, but some caution needs to be expressed in making this claim.

In his responses to Stivers and Rossano (2010), on why there may be delays longer than predicted by Sacks et al. (1974), or even no response at all, Schegloff (2010) makes the point that participants in these conversations may be in “continuing states of incipient talk” (Schegloff and Sacks, 1973). Couper-Kuhlen (2010) makes a similar point, reminding us of Goffman's distinction between “focused” and “non-focused” gatherings, with the latter displaying a lack of “tightly organized exchange of doings,” and thus perhaps less urgency to produce second pairs parts or, one might add, less urgency to avoid extended silences between turns. Such situations might include “members of a household in their living rooms, employees who share an office, passengers together in an automobile” (Schegloff and Sacks, 1973, pp. 324–325), or, one might further add, old ladies sitting for hours on the porch of a house in the heat of the day. Indeed, the examples from white Australian couples' conversation, where at times similarly long gaps between turns to those in the Garrwa conversations were found, provide some support for Schegloff's and Couper-Kuhlen's observations. In reference to some of the long silences in her data, Jefferson (1989) muses that there may be a “‘relaxation” of certain “rules” among intimates’ (p. 192). She is referring here specifically to a speaker completing another speaker's turn, where that other speaker is a spouse or sibling. Something similar may be going on in these Garrwa conversations, namely a relaxation of rules amongst intimates where gaps are not minimized. The women in our data grew up together, so are more like family than close friends (in fact, Hilda and Daphne are sisters-in-law); they have lived in small communities in close proximity to each other for many years. Long silences between turns—gaps that do not transform into lapses—are not only tolerated, but are common. In addition, these women have little to do. They sit around for hours at a time, passing the time of day in conversation. Conversations involving intimates in familiar surroundings, with a lack of pressure to talk may in fact be at least as pertinent as cultural difference in accounting for expanded transition spaces.

More specific and local reasons for delays in responding, as some of the examples in the current study suggest, may be “disengagement or lack of attention” (Levinson, 2010), the “activity-occupied withdrawal” that Goodwin (1981) notes, such as brushing one's hair, or seeking something in a handbag. Our analyses also point to the possibility that there is less urgency to respond if a next speaker has not been selected than if they have. Further, but generally less amenable to verification, if a next speaker lacks the knowledge to respond, it is likely that a response may be delayed, or a non-complying response may transpire, or there may be no response at all. Such local factors may then be in play with the wider overall structure of the encounters noted above, and when these local conditions occur during a “non-focused gathering,” the frequency of longer gaps between turns may increase.

We do not feel confident to claim that these longer gaps can be explained by culture or ethnicity, even if there is growing evidence that there may be a greater tolerance (or at least occurrence) of longer gaps in some cultures or language groups than others. If gaps are indeed more frequent in some cultures than others, such as Garrwa (and other Indigenous Australian languages) or ≠Akhoe Hai||om (Hoymann, 2010), spoken in Namibia, it may be that the more traditional life style in very remote areas with relatively little contact to the modern, industrial world does not fully explain the slower pace of conversation, but rather that in such traditional or semi-traditional societies or communities, the people are more likely to live their lives at a slower tempo, in more “non-focused gatherings,” and less “tightly organized exchanges of doings.” In the conversations we have examined, the old women sit around for hours. They have few appointments to meet, nowhere much to go, little pressure to do anything. They are in familiar surroundings, where they live in close proximity to and know everyone else, as in an extended family. But also they are sitting outside, where people are passing, there are things to watch and notice. Much of the time they are loosely engaged with each other. Life's pace is slow. Conversation is slow. Nevertheless, when the occasion demands, they are perfectly capable of fast-paced conversation, and can provide responses with no gap and no overlap.

As Schegloff (2000) has noted,

nothing special rests on the “one-at-a-time” proposal. Should a compelling demonstration of a different way of organizing participation in conversation be provided, it would allow us to seek a more general account that could subsume both one-at-a-time and its alternative(s) as special cases

(p. 47).

The materials presented here in many ways suggest no more than was already accounted for in Sacks et al. (1974) under rule 1c, and some subsequent notes on this rule on how discontinuous talk emerges. If no speaker selects next speaker, no potential next speaker self-selects, and current speaker chooses not to continue, then a gap develops. Such gaps may occur more or less regularly, and develop into shorter or longer gaps, and extend further or less far toward lapses. In some conversations, with some participants, in some low-pressure situations, and perhaps even in some cultures, it may be the case that the option not to continue under 1c is exercised more regularly, and that once the transition space has passed, the floor opens up to anyone to self-select as next speaker. This could even be posited as a “lowest order rule” of turn-taking: if current speaker chooses not to continue speaking under rule 1c, then after closure of the regular transition space, any speaker may self-select at any time, first speaker to self-select gaining rights to speak. Where the option not to continue speaking is regularly exercised, then the gross observation for “one-at-a-time” will not hold as a recurrent feature of such talk in such circumstances.

There is one final point to be made. We have found that these Garrwa speakers are quite capable of distributing their conversational turns in just the way that Sacks et al. (1974) described, with next speaker selection, self-selection if a next speaker hasn't been selected, and continuation by the current speaker if no other speaker has self selected. The turns in these Garrwa conversations are constructed in units that are identifiable as TCUs, and there is orientation to TRPs, albeit often extended ones. Apart from Sidnell's (2001) study of Caribbean Creole English and Tanaka's (2000) study of Japanese, there have been few studies about the rules of turn-taking of languages other than English, particularly of languages (and cultures) very different from English such as Garrwa, that have shown that the fundamental rules of turn-taking are followed as Sacks, Schegloff, and Jefferson describe. Yet it is through studies such as these that we are able to enrich our understanding of what is fundamental about human social interaction.

### Conflict of interest statement

The authors declare that the research was conducted in the absence of any commercial or financial relationships that could be construed as a potential conflict of interest.

## Footnotes

^1^Since 1974, the notion of what constitutes a turn-constructional unit has developed further, see for example Schegloff (1996) and Ford and Thompson (1996).

^2^Where a “normal” transition-relevance place begins and ends is not often discussed explicitly in the literature, but the normal, or default space has been declared to be one “beat of silence,” i.e., the time it takes to say a single syllable at normal rate (Jefferson, 1984; Schegloff, 2000). Wells and Macfarlane (1998) suggest that it extends from a final, turn-ending projecting accent in a TCU to the onset of a next speaker's talk, which may typically be about two beats, or 0.2 s.

^3^While Kendrick and Torreira (2014) reported that dispreferred responses occur more commonly than preferred responses after a longer gap of silence, they also found that the most frequently occurring dispreferred and preferred responses occur after gaps of a similar length.

^4^Whilst lapses do not occur after “current speaker selects next” has been applied, it can be the case that gaps open up, and in these gaps it may be that, in the absence of a response by the selected speaker beyond the “normal” transition space (cf. Stivers and Rossano, 2010), another speaker may respond (Stivers and Robinson, 2006). In the cases these authors present, the gaps are mostly less than a second before a non-selected speaker responds. In some other cases, the selected speaker may be engaged in some other activity, contributing to the non-response. A central point the authors make is that there are two competing preferences at work when “current speaker selects next” is applied: one for the speaker so selected to speak, and a second for the action implemented by the current speaker's turn, e.g., a question, to be responded to, e.g., with an answer (if necessary by another speaker if the selected speaker cannot or does not answer). They say in relation to these two competing preferences that “the rule (i.e., 1a) is incrementally relaxed, suggesting an additional order of organization exists past the TRP which is laminated on top of the existing rule” (p. 391).

^5^We are not sure how one can determine that “rounds” of possible self-selection may develop (cf. Wilson and Zimmerman, 1986). An alternative possibility is that once the transition place has passed, and no next speaker has been selected under rule 1a, any speaker can self-select. This is, of course, a matter for empirical investigation, and is beyond the scope of this paper. However, cases such as the following (taken from the first author's data) raise interesting questions.
1  Liz:   =Oh;= that's good.
2         (6.5)
3  Mel:   °°(whoohh hehh)°°
4         (8.6)
5  Mel:  ->    [Tom-] (.) [Tommy-]
6  Liz:  ->    [D'ju] [D'ju wa-]
7  Liz:        Sorry-. Mel d'yu wan' that o nion.= bicoz-
8              (0.7) °I- (.) > don' thing I'll< (.) >be
9              able ta< digest it.
Liz and Mel are at the dinner table. After line 1, a silence ensues, grows, and becomes a lapse, though there is a sigh after six-and-a-half seconds. After no talk for about 16 s, both start to speak simultaneously. Both drop out after one beat of talk, there's a micro-pause, both begin to talk again with identical starts, this time they go on for two beats, and again both drop out. Liz then gets the floor at the third attempt, starting with an apology. The question is what prompted such precision timing in their starts after such a long silence. Preliminary findings by the first author suggest that some body coordination occurs before such post-lapse simultaneous starts. For the arguments of direct relevance to the current paper, such instances would suggest that after a certain length of silence, any speaker may start at any time. Whether that is the case immediately after closure of the transition space, or after a second or so (cf. Jefferson, 1989), or after some other metric remains to be determined. If this turns out to be the case, it would mean that the priority given to self-selection (rule 1b) over current speaker continues (rule 1c) would at some point be superseded.

^6^We acknowledge the shortcomings of working solely with audio recorded data. The Borroloola recordings were originally made for linguistic analysis, and only later used for conversation analysis. For this reason, we use them only when there are no good illustrations of the points we are making from the video data. Given the relative dearth of Conversation Analytic studies on endangered, non-Western and non-industrialized language groups, we feel justified in including some audio-only data.

^7^To preserve anonymity, names of participants have been changed.

^8^A coolamon is a vessel made of bark or wood for carrying water, babies etc.

^9^Other activities in the Robinson River conversation which either were seen to accompany talk, or in our judgment could do so, were: shaking an asthma inhaler, waving away flies, wiping away sweat with a towel, rolling small objects on the lap or thigh, putting objects into a handbag, scratching, brushing hair, and opening or closing a bottle, as well as peering out into the surroundings.

^10^It is possible that Daphne is responding late to the girl's turn in line 1223, but even were this so, the precision timing of Daphne's “when,” latched to a point of possible completion, remains.

^11^It is unclear to us who the “mother” is who is being referred to here, and why she is being referred to. It is a common practice in Aboriginal communities for reference to persons to be indirect or vague (Blythe, 2009; Garde, 2003). This phenomenon can be explained, at least in part, because they live in small communities in which everyone knows everyone else, but also because there are kinship related taboos on naming certain persons directly. Be that as it may, following Tina's repair initiating “Yindi” in line 98, there is a sequence in which the reference appears to be resolved to the satisfaction of Tina. The point we are making in this extract about turn-taking practices following Sacks et al. (1974) rules holds despite the reference to “mother” remaining unclear.

^12^The vagueness of “they used to” in response to a “who” question in line 1006 reflects a common practice in Indigenous Australian talk of vague person reference (eg., Garde, 2003; Blythe, 2013). This may in part have to do with the small, close-knit communities in which everyone knows everyone else. It may also have to do with taboos, where naming of certain kin is forbidden (Blythe, 2013). This issue is beyond the scope of this paper.

^14^Unfortunately these are all in the Borroloola audio-only data, so they cannot be checked for what the participants were doing during the silences.

^15^Gardner (2010) also found that 45% of questions were never answered, which is higher than any of the ten languages investigated by Stivers et al. (2009), which was Korean at 36%.

## Abbreviations

ABL: ablative
ACC: accusative
ALL: allative
BARRI: a discourse particle
CONJ: conjunction
DAT: dative
DEM: demonstrative
DS: different subject (switch reference marker)
ERG: ergative
FUT: future
HAB: habitual
IMP: imperative
INTENS: intensifier
KANYI: verbal morpheme (yet to be labeled)
LOC: locative
NA: a discourse particle
NEG: negative particle
PA: past tense
PURP: purposive
WA: grammatical morpheme (yet to be labeled)
1sg: first person singular
2sg: second person singular
1duIncl: first person dual inclusive (you and me)
3du: third person dual
1plncl: first person plural inclusive
1plExcl: first person plural exclusive
3pl: third person plural.
